# Effect of Laser Surface Texturing on Bond Strength and Mechanical Properties of 3Y and 5Y Zirconia

**DOI:** 10.3390/ma19020410

**Published:** 2026-01-20

**Authors:** Eun-Suk Lee, Min-Gyu Song, Yoon-Hyuk Huh, Chan-Jin Park, Lee-Ra Cho, Kyung-Ho Ko

**Affiliations:** Department of Prosthodontics and Research Institute of Oral Science, College of Dentistry, Gangneung-Wonju National University, Gangneung 25457, Republic of Korea; prosthostone@naver.com (E.-S.L.); smg2763@naver.com (M.-G.S.); vino@gwnu.ac.kr (Y.-H.H.); doctorcj@gwnu.ac.kr (C.-J.P.); lila@gwnu.ac.kr (L.-R.C.)

**Keywords:** zirconia, surface treatment, shear bond strength, flexural strength

## Abstract

This study evaluated the influence of various surface treatments on the bonding performance and mechanical behavior of zirconia, with particular emphasis on the effect of laser surface texturing (LST) compared with conventional 10-methacryloyloxydecyl dihydrogen phosphate (10-MDP) and airborne particle abrasion (APA) methods. Two zirconia compositions, 3 mol% yttria-stabilized tetragonal zirconia polycrystal (3Y-TZP) and 5 mol% partially stabilized zirconia (5Y-PSZ), were subjected to four surface treatment protocols: as-milled, 10-MDP, APA, and LST (*n* = 12). Shear bond strength (SBS) to titanium and biaxial flexural strength (BFS) of zirconia were measured. Surface morphology, failure mode, and phase composition were analyzed using scanning electron microscopy (SEM), energy-dispersive spectroscopy (EDS), and X-ray diffraction (XRD). Data were analyzed with two-way ANOVA and Tukey’s post hoc test (α = 0.05), and the reliability of flexural strength was assessed using Weibull analysis. Surface treatment significantly affected SBS (*p* < 0.05). The LST groups exhibited the highest SBS values and a higher proportion of mixed failures, whereas other groups predominantly showed adhesive failures. However, LST-treated specimens, particularly 5Y-PSZ, showed reduced BFS. XRD confirmed phase stability, although localized microstructural changes were observed after LST. LST enhanced the zirconia–titanium interfacial bond strength and promoted mixed failure modes; however, this improvement was accompanied by a reduction in flexural strength, particularly in 5Y-PSZ.

## 1. Introduction

Zirconia is widely employed as a restorative material in contemporary dentistry because of its favorable biocompatibility, chemical inertness, and superior mechanical strength [1,2]. Among the various formulations, 3 mol% yttria-stabilized tetragonal zirconia polycrystal (3Y-TZP) exhibits high flexural strength and fracture toughness, whereas 5 mol% partially stabilized zirconia (5Y-PSZ) offers enhanced translucency owing to its higher cubic phase content [3,4]. Despite these advantages, both materials remain polycrystalline ceramics with inherently low surface reactivity, which makes achieving durable adhesion to cements a persistent clinical challenge [5,6].

To enhance adhesion, different surface treatment strategies have been developed [7,8,9,10,11]. 10-methacryloyloxydecyl dihydrogen phosphate (10-MDP) promotes stable phosphate–zirconium bonding through ionic and hydrogen interactions with surface hydroxyl groups, producing Zr–O–P linkages and an adsorbed molecular layer that increases surface energy and improves wettability [8,12,13]. In contrast, airborne particle abrasion (APA) with alumina particles modifies the surface topography by generating micro-irregularities and introducing localized compressive stress, which enhances micromechanical interlocking. However, excessive abrasion may lead to surface damage or phase transformation from the tetragonal to monoclinic phase, potentially weakening the material. Although both 10-MDP and APA improve bond strength, debonding of zirconia restorations continues to be reported clinically, likely reflecting the limited durability of chemical bonds and the instability of micromechanical retention [7,14,15]. These limitations have prompted interest in physical approaches such as laser surface texturing.

Laser surface texturing (LST) has recently emerged as a physical alternative for surface modification. This technique employs high energy laser irradiation to create controlled micro patterns on the zirconia, increasing roughness and surface energy without introducing chemical residues or potential phase transformation. LST is expected to combine the micromechanical advantages of APA with the chemical stability of zirconia, potentially improving bonding reliability. However, the localized thermal energy generated during laser processing may modify grain structure or induce residual stresses that can adversely affect mechanical strength [16].

Although numerous studies have evaluated the bonding performance of zirconia after various surface treatments, limited evidence exists on how these treatments simultaneously affect mechanical integrity. In particular, the balance between improved adhesion and potential degradation in flexural strength remains unclear, especially when comparing 3Y-TZP and 5Y-PSZ with their distinct crystalline compositions.

Therefore, the purpose of this study was to analyze the surface characteristics of zirconia before and after LST treatment and to comprehensively evaluate the effects of different surface treatments on bonding performance and mechanical properties. The null hypotheses were that (1) no significant differences would be found in shear bond strength (SBS) and biaxial flexural strength (BFS) between the two zirconia compositions and (2) no significant differences would be observed among the surface treatment methods—as-milled, 10-MDP, APA, and LST.

## 2. Materials and Methods

Two zirconia compositions were evaluated: 3 mol% yttria-stabilized tetragonal zirconia polycrystal (3Y-TZP; LUXEN Zr, Dentalmax, Cheonan, Republic of Korea) and 5 mol% partially stabilized zirconia (5Y-PSZ; LUXEN 5 G Smile, Dentalmax, Cheonan, Republic of Korea).

### 2.1. Surface Treatment Protocols

For the shear bond strength (SBS) test, four surface treatment protocols were applied to each zirconia type: as-milled (A), 10-MDP primer (M), airborne particle abrasion (P), and laser surface texturing (L). For the biaxial flexural strength (BFS) test, three groups (A, P, L) were evaluated, as the 10-MDP primer treatment was excluded because it represents a chemical, rather than a mechanical, surface modification and is not expected to influence flexural strength.

A group received no post-milling modification. M group was treated with a phosphate-containing primer (G-Multi Primer, GC Corp., Tokyo, Japan). P group was abraded with 50 µm aluminum oxide particles at 2.5 bar pressure for 10 s [17]. L group was subjected to a laser surface texturing system (STM, Ceramic Technology Co., Seoul, Republic of Korea) in accordance with the manufacturer’s guidelines prior to sintering, thereby generating a controlled microtexture pattern on the zirconia surface.

The L group specimens underwent surface texturing at the pre-sintering stage and were subsequently sintered in a ceramic furnace (Duotron Pro S-600, Add-in, Bucheon, Republic of Korea) according to the manufacturer’s protocol. In contrast, the A, M, and P groups were fully sintered first and then subjected to their respective surface treatments. The firing schedule included heating at 9 °C/min to 1000 °C with a 10-min hold, followed by heating to 1530 °C at 3.5 °C/min with a 120 min hold and controlled cooling at −8 °C/min.

### 2.2. Surface Roughness Measurement

Surface roughness of the zirconia specimens was measured using a portable profilometer (TR-200, TIME Group, Beijing, China). Prior to measurement, the device was positioned perpendicular to the specimen surface to ensure accurate contact. The measurement length was set to 0.8 mm, with a cutoff length of 0.25 mm. For each specimen (*n* = 12), three different locations were measured, and the arithmetic mean of these values was calculated and used as the representative surface roughness. The surface roughness parameter evaluated was the average roughness (Ra, µm).

### 2.3. Shear Bond Strength Test

Rectangular zirconia specimens (12 × 14 × 3 mm) were fabricated in accordance with ISO 29022 [18] (*n* = 12). A cylindrical titanium rod was bonded to the center of each specimen using a self-adhesive resin cement (RelyX U200, 3 M ESPE, Seefeld, Germany). Prior to bonding, titanium surfaces were abraded with 50 µm alumina at 2 bar pressure for 30 s [19] and ultrasonically cleaned in distilled water for 5 min.

Bonded specimens were stored at room temperature (25 °C) for 24 h and then tested in a universal testing machine (Instron 3345, Instron Corp., Norwood, MA, USA) at a crosshead speed of 1.0 mm/min. The shear load was applied perpendicularly to the bonding interface until failure occurred.

### 2.4. Biaxial Flexural Strength Test

Disc-shaped zirconia specimens (12 mm diameter, 2 mm thickness) were prepared in accordance with ISO 6872 [20] (*n* = 12). Each specimen was placed on three supporting steel balls (10 mm span) and loaded centrally using a flat-ended indenter (1.6 mm diameter) in a universal testing machine (RB 302 ML, R&B Inc., Daejeon, Republic of Korea) at a crosshead speed of 1.0 mm/min. Fracture loads were converted to biaxial flexural strength (MPa). Weibull analysis was performed to determine the reliability of each group.

### 2.5. Surface Characterization

Representative specimens from each group were analyzed using field emission scanning electron microscopy (FE-SEM; JSM-7610F Plus, JEOL Ltd., Tokyo, Japan). Surfaces were sputter-coated with platinum to a thickness of 10 nm and observed at 10 kV acceleration under magnifications of ×100 and ×20,000.

Elemental composition was assessed by energy-dispersive X-ray spectroscopy (EDS; AztecLive, Oxford Instruments, Abingdon, UK) attached to the SEM to identify major elements such as aluminum (Al), zirconium (Zr), and oxygen (O).

Phase composition was examined using X-ray diffraction (XRD; D8 DISCOVER, Bruker AXS GmbH, Karlsruhe, Germany). Scanning was conducted from 20° to 80° (2θ) at a step size of 0.02° and a scan speed of 2 °/min.

### 2.6. Statistical Analysis

A power analysis was performed through a pilot study (G*Power 3.1, Düsseldorf, Germany) and the sample size was determined to be 12 samples per group. Data were analyzed using SPSS software (Version 26.0; IBM Corp., Armonk, NY, USA). The Shapiro–Wilk test confirmed normality, and Levene’s test verified homogeneity of variance. Two-way ANOVA was performed to assess the effects of zirconia type and surface treatment, followed by Tukey’s post hoc test (α = 0.05) for SBS, surface roughness and BFS. And weibull parameters were calculated from BFS data to assess reliability.

## 3. Results

**Figure 1:**
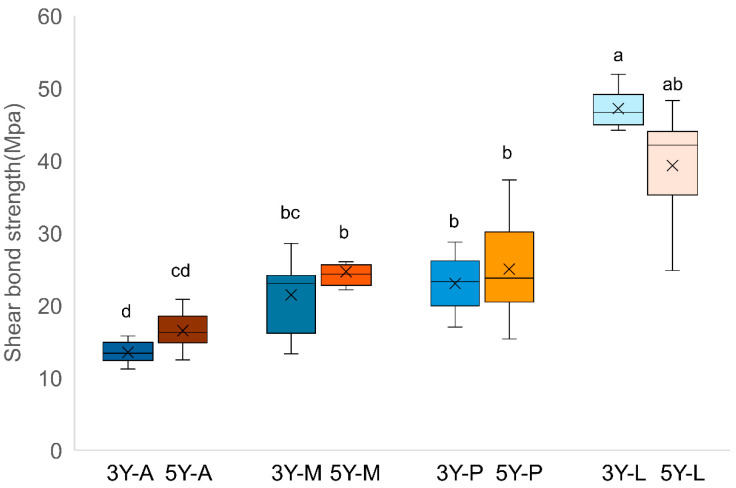
Box plot of shear bond strength for each experimental group. Different lowercase letters above the boxes indicate statistically significant differences among groups according to Tukey’s post hoc test (*p* < 0.05). A: As-milled, M: 10-methacryloyloxydecyl dihydrogen phosphate, P: Airborne particle abrasion, L: laser surface texturing.

The SBS results are summarized in Figure 1 and Table 1. Both zirconia types showed a similar trend across surface treatments. Two-way ANOVA showed significant differences among surface treatments (*p* < 0.05). The LST-treated groups (3Y-L, 5Y-L) exhibited the highest mean SBS values, which were significantly greater than those of the other groups (*p* < 0.05). The 3Y-M and 3Y-P groups showed higher SBS than the 3Y-A (*p* < 0.05). A comparable pattern was observed in 5Y-PSZ, where 5Y-L exhibited the highest SBS (*p* < 0.05), and 5Y-P showed higher values than 5Y-A (*p* < 0.05), while 5Y-M was not significantly different (*p* > 0.05). Two-way ANOVA demonstrated that the effect of zirconia type on SBS was not significant (*p* > 0.05). Nevertheless, two-way ANOVA identified a significant material-by-treatment interaction for SBS (*p* < 0.05). Therefore, simple-effects comparisons were reported within each zirconia type. Surface treatment remained significant overall (*p* < 0.05).

Failure-mode analysis revealed exclusively adhesive failures in the A, M, and P groups, whereas the L group showed a higher incidence of mixed failures, reflecting greater interfacial resistance. Specifically, 3Y-L exhibited 8 adhesive and 4 mixed failures, and 5Y-L exhibited 9 adhesive and 3 mixed failures.

**Figure 2:**
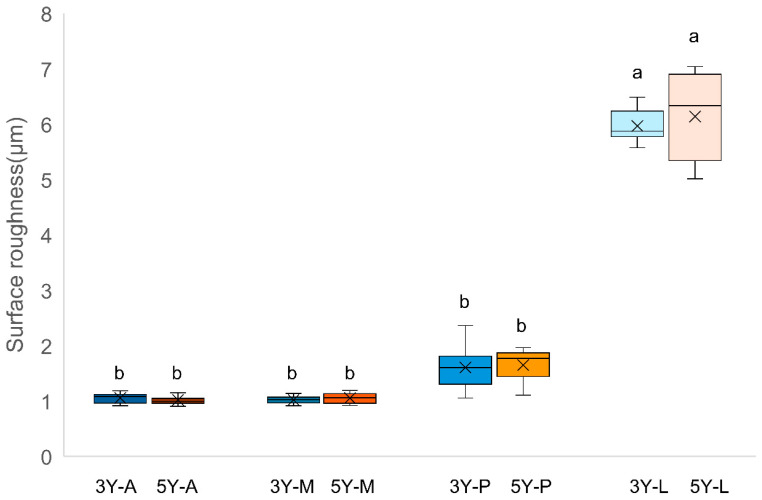
Box plot of surface roughness for each experimental group. Different lowercase letters above the boxes indicate statistically significant differences among groups according to Tukey’s post hoc test (*p* < 0.05). A: As-milled, M: 10-methacryloyloxydecyl dihydrogen phosphate, P: Airborne particle abrasion, L: laser surface texturing.

Surface roughness results are presented in Figure 2 and Table 1. Two-way ANOVA showed no significant interaction between zirconia type and surface treatment (*p* > 0.05); however, surface treatment alone significantly affected roughness (*p* < 0.05). L group specimens significantly increased surface roughness compared with all other treatments (*p* < 0.05). A, M, and P groups showed similar Ra values (*p* > 0.05). These findings confirmed that laser surface texturing produced a distinct topographic modification compared with conventional methods.

**Figure 3:**
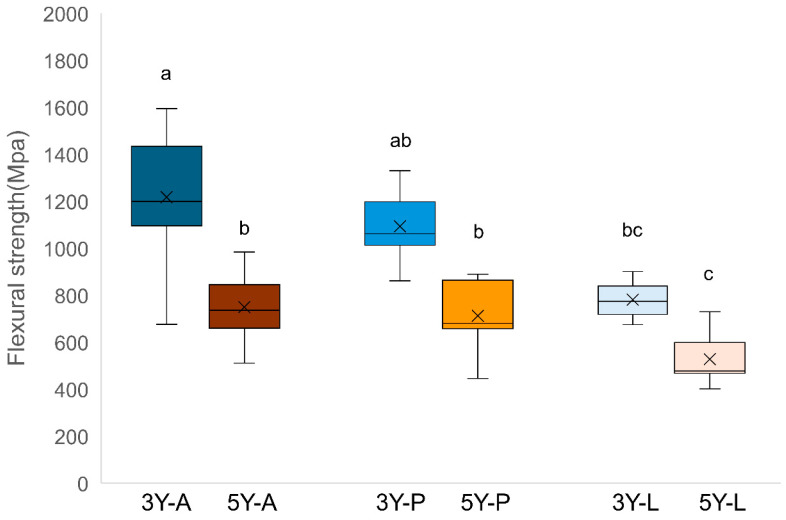
Box plot of biaxial flexural strength for each experimental group. Different lowercase letters above the boxes indicate statistically significant differences among groups according to Tukey’s post hoc test (*p* < 0.05). A: As-milled, P: Airborne particle abrasion, L: laser surface texturing.

The BFS results are shown in Figure 3 and Table 1. In both zirconia types, flexural strength decreased following LST treatment. The 3Y-A group exhibited the highest mean BFS, followed by 3Y-P and 3Y-L (*p* < 0.05). In the 5Y-PSZ series, all groups presented lower BFS values than their 3Y counterparts (*p* < 0.05), and 5Y-L exhibited the lowest mean strength (*p* < 0.05).

**Figure 4:**
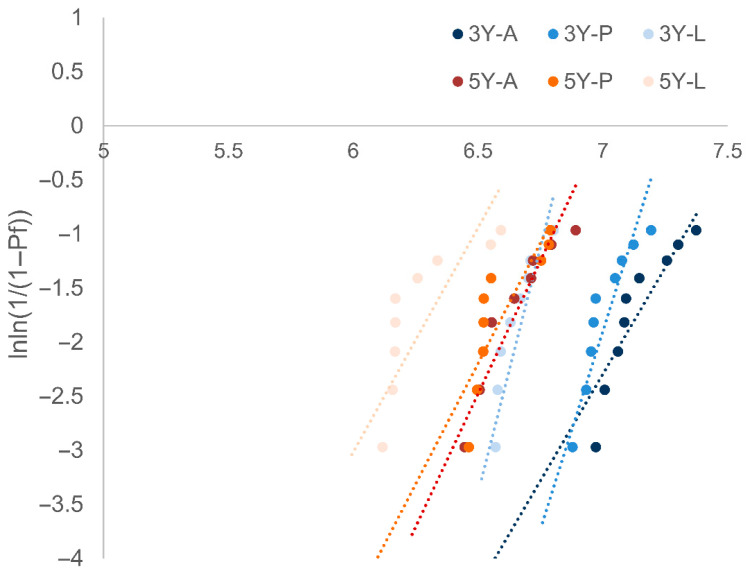
Weibull plot of biaxial flexural strength for each group. The x-axis represents the measured flexural strength (MPa), and the y-axis represents the transformed Weibull cumulative probability [lnln(1/(1 − P))]. A: As-milled, P: Airborne particle abrasion, L: laser surface texturing.

Weibull plots (Figure 4) showed the steepest slope for 3Y-L. Although the 3Y-L group exhibited the lowest mean flexural strength, its steep Weibull slope indicated consistent strength distribution within the group. The 5Y groups displayed shallower slopes, suggesting greater variability and reduced reliability.

**Figure 5:**
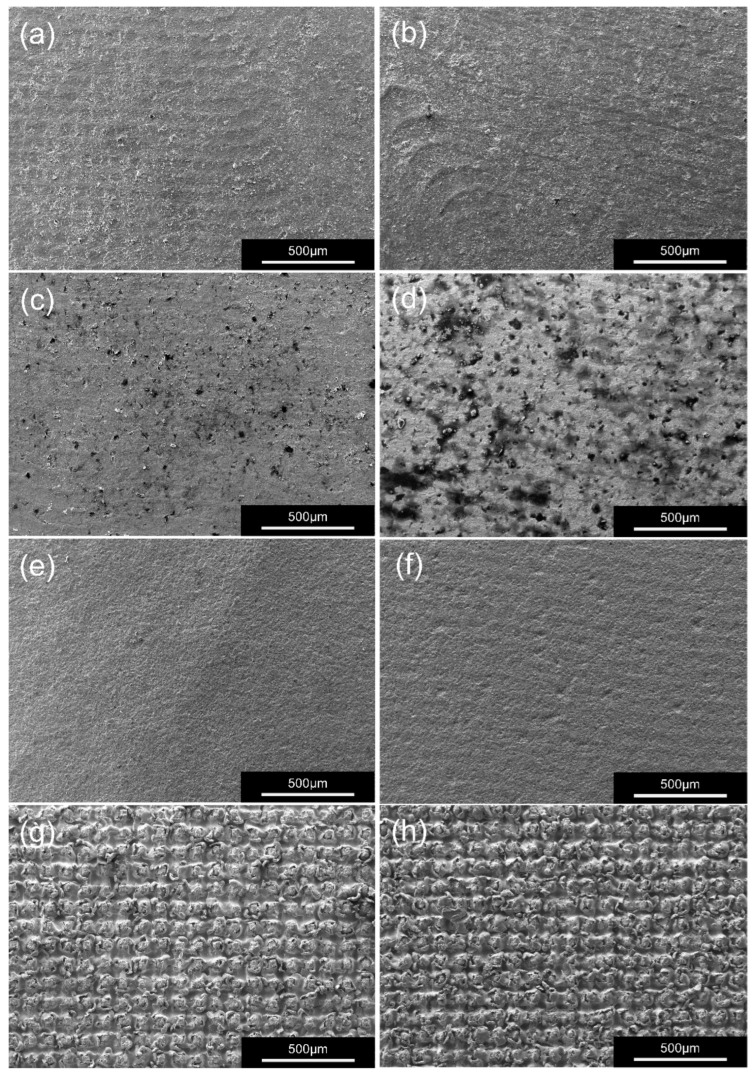
Representative SEM images at 100× magnification showing the surface morphology of zirconia specimens after different surface treatments. (**a**) 3Y-A: as-milled 3Y-TZP, (**b**) 5Y-A: as-milled 5Y-PSZ, (**c**) 3Y-M: 10-MDP-treated 3Y-TZP, (**d**) 5Y-M: 10-MDP-treated 5Y-PSZ, (**e**) 3Y-P: air abrasion-treated 3Y-TZP, (**f**) 5Y-P: air abrasion-treated 5Y-PSZ, (**g**) 3Y-L: laser surface textured 3Y-TZP, (**h**) 5Y-L: laser surface textured 5Y-PSZ.

**Figure 6:**
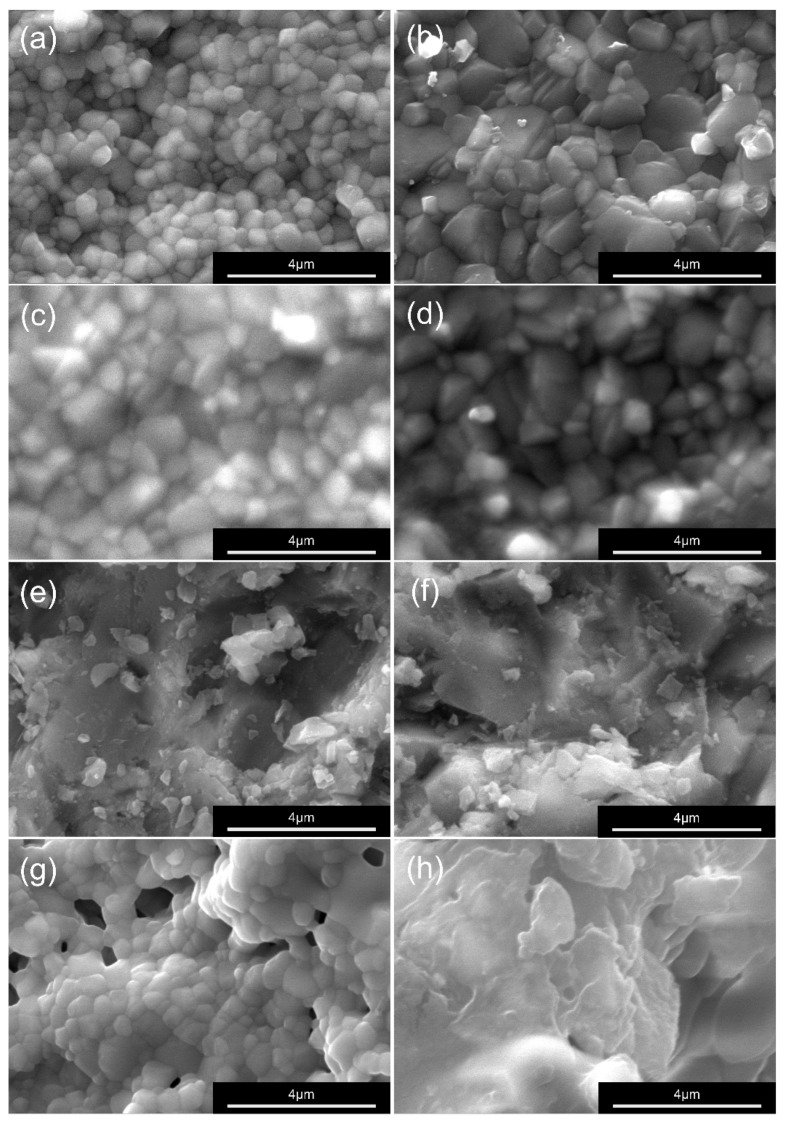
Representative SEM images at 20,000× magnification showing the surface morphology of zirconia specimens after different surface treatments. (**a**) 3Y-A: as-milled 3Y-TZP, (**b**) 5Y-A: as-milled 5Y-PSZ, (**c**) 3Y-M: 10-MDP-treated 3Y-TZP, (**d**) 5Y-M: 10-MDP-treated 5Y-PSZ, (**e**) 3Y-P: air abrasion-treated 3Y-TZP, (**f**) 5Y-P: air abrasion-treated 5Y-PSZ, (**g**) 3Y-L: laser surface textured 3Y-TZP, (**h**) 5Y-L: laser surface textured 5Y-PSZ.

Representative SEM images (Figure 5 and Figure 6) showed distinct surface morphologies among treatments. P group produced irregular microdefects and residual alumina particles, whereas 10-MDP groups exhibited smoother surfaces with localized chemical deposits. In contrast, L group specimens displayed uniform lattice-like microtextures with clear grain boundaries.

EDS analysis identified zirconium, oxygen, and yttrium as the main elements in all groups (Table 2). Oxygen content was slightly higher in the L groups, while yttrium content was lowest in 3Y-L.

**Figure 7:**
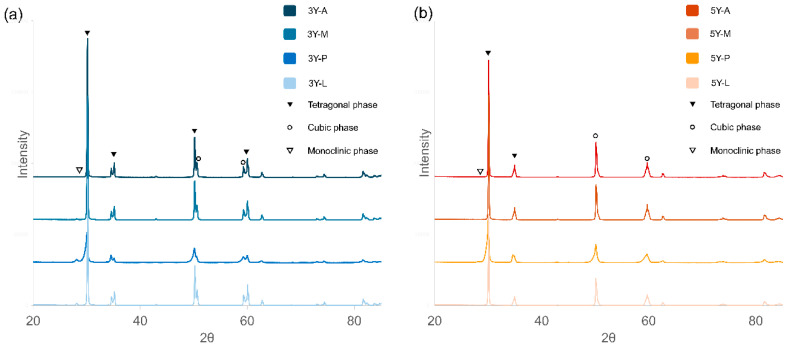
X-ray diffraction (XRD) patterns of zirconia specimens according to material type and surface treatment. (**a**) 3Y-TZP groups: all patterns show dominant tetragonal phase; monoclinic peaks were observed in the P group. (**b**) 5Y-PSZ groups: all patterns show t-phase dominance with lower peak intensities, and the P group exhibited distinct monoclinic peaks. The diffraction patterns were used for qualitative phase identification and comparison among the groups, and the symbols indicate characteristic diffraction peaks for each phase. A: As-milled, M: 10-methacryloyloxydecyl dihydrogen phosphate; P: Airborne particle abrasion; L: laser surface texturing.

XRD analysis (Figure 7) confirmed dominant tetragonal peaks in all 3Y-TZP groups. Minor monoclinic phase transformation was detected in APA-treated specimens. In 5Y-PSZ, monoclinic peaks were more distinct in the P group, whereas the L group maintained sharper tetragonal peaks, indicating better phase stability after laser processing.

## 4. Discussion

This study evaluated the effects of zirconia composition and surface treatment on bonding performance and mechanical behavior. Surface treatment exerted a significant effect on SBS, while zirconia composition showed no independent influence. In contrast, both factors significantly affected BFS. Therefore, the null hypotheses were partially rejected.

The results indicated that both zirconia composition and surface treatment parameters markedly influenced bonding performance. This relationship underscores the combined roles of surface chemistry and microstructural configuration in determining interfacial adhesion. Notably, 3 mol% yttria-stabilized tetragonal zirconia polycrystal (3Y-TZP) and 5 mol% partially stabilized zirconia (5Y-PSZ) differ fundamentally in phase composition, transformation toughening capability, and intrinsic strength, which should be considered when interpreting their distinct responses to surface modification in both mechanical and clinical contexts. Specimens treated with 10-methacryloyloxydecyl dihydrogen phosphate (10-MDP) or airborne particle abrasion (APA) showed significantly higher shear bond strength than the as-milled controls, reflecting the effects of chemical bonding and micromechanical retention. Likewise, the 5Y-M and 5Y-P groups, with their higher cubic-phase content, exhibited slightly greater bond strength than the corresponding 3Y groups. This trend may be explained by the stabilization of the cubic phase, which resists monoclinic transformation and modulates surface hydroxylation, thereby promoting a more favorable chemical interaction with 10-MDP [21,22,23]. These findings align with previous reports demonstrating that 10-MDP forms stable Zr–O–P linkages and an adsorbed molecular layer that enhances interfacial reactivity [24]. In the present study, this chemical interaction appeared more pronounced in the 5Y-PSZ specimens, which exhibited localized surface deposits and smoother interfacial regions under SEM observation.

Although no statistically significant difference was observed between zirconia compositions, scanning electron microscopy (SEM) analysis revealed clear morphological distinctions among the treated surfaces. Both 3Y-P and 5Y-P specimens exhibited residual alumina particles and localized surface irregularities after airborne particle abrasion, indicating that the higher cubic-phase content of 5Y-PSZ limited the effectiveness of mechanical modification compared with 3Y-TZP. Laser surface texturing (LST) produced the greatest surface roughness through controlled microtexturing, which likely enhanced micromechanical interlocking at the interface. This was further supported by the higher incidence of mixed failure modes in the LST groups, suggesting a more robust and integrated zirconia–titanium bonding interface compared with conventional treatments. In contrast, the 10-MDP and APA treatments generated only subtle topographic alterations, consistent with their limited ability to induce structural changes [21,25].

Although both zirconia compositions were subjected to identical pre-sintering LST conditions, the 3Y-L group demonstrated higher shear bond strength than the 5Y-L group. X-ray diffraction (XRD) analysis revealed a distinct tetragonal peak in 3Y-TZP. Phase transformation was observed in the 3Y-P group, while minimal phase transformation was observed in the 3Y-L group after laser texturing and sintering. In contrast, the predominantly cubic structure of 5Y-PSZ appeared less responsive to laser patterning, resulting in a comparatively less retentive surface texture [22,26,27]. Collectively, these findings indicate that LST promotes adhesion primarily through microstructural modulation rather than chemical activation, and its effectiveness is intrinsically material-dependent.

The results of surface characterization further substantiated these interpretations. SEM revealed that LST generated uniform lattice-like microtextures with well-defined grain boundaries, whereas APA created irregular defects and left residual alumina particles on the surface. From a clinical perspective, these findings highlight a key distinction between the controlled, particle-free nature of laser surface texturing and the defect- and contamination-prone characteristics of airborne particle abrasion. Energy-dispersive spectroscopy (EDS) detected a slightly higher oxygen content in the L group specimens, suggesting increased surface oxidation and higher surface energy favorable for chemical interaction with resin. XRD patterns displayed stable tetragonal peaks in 3Y-L and well-defined peaks in 5Y-L, indicating that laser processing did not cause any deleterious phase transformation.

In the BFS test, the 3Y-A group showed the highest flexural strength, whereas the 3Y-L group exhibited a reduction of nearly one-third. Despite this reduction, the remaining flexural strength values of LST-treated zirconia, particularly in 5Y-PSZ, remained within ranges reported for clinically used monolithic translucent zirconia, although with a reduced mechanical safety margin in high-load indications. A similar pattern was observed in the 5Y series, where the 5Y-L specimens demonstrated the lowest strength values. Notably, the 3Y-P and 5Y-P groups responded differently to APA. The 3Y-TZP specimens exhibited localized tetragonal-to-monoclinic transformation and compressive stress–induced toughening, whereas the predominantly cubic 5Y-PSZ showed minimal transformation. These microstructural differences may account for the distinct adhesive behavior between the two zirconia compositions under identical APA conditions.

In contrast to the SBS findings, the reduction in flexural strength after LST indicates that enhanced adhesion was accompanied by compromised mechanical integrity. Because laser surface texturing was applied prior to sintering, the observed strength reduction should be interpreted as a combined effect of surface modification and altered thermal history rather than as a purely geometric surface phenomenon. The localized thermal energy generated during laser processing may have introduced residual stresses or initiated microcracks within the pre-sintered matrix. Additionally, heat-induced grain coarsening and premature thermal exposure could further reduce strength [28,29]. From this perspective, LST involves a dual thermal process: micro-patterns are inscribed during the pre-sintering stage, when zirconia undergoes partial densification, and the material is subsequently exposed to additional heat cycles during final sintering. Previous studies have shown that post-sintering treatments such as glazing or reheating promote grain growth and residual stress accumulation, both of which are associated with reduced mechanical strength [30,31]. These findings suggest that the overlapping heat exposure during LST and sintering may produce comparable thermal effects, explaining the strength reduction observed in this study.

Taken together, these results suggest that LST achieves a balance between improved adhesion and mechanical stability. In this context, the present study intentionally evaluated the outcome of a clinically applicable commercial LST workflow rather than optimization or comparison of individual laser parameters, focusing on resultant surface characteristics and material performance. Accordingly, laser effects were interpreted based on observable morphological and mechanical outcomes, in line with a result-oriented approach rather than parameter-driven laser optimization. Whereas 10-MDP enhanced bonding without affecting strength and APA increased adhesion at the expense of introducing surface flaws, LST produced the greatest micromechanical retention but also caused a noticeable reduction in flexural strength. Therefore, the clinical applicability of LST should be viewed as a balance between interfacial adhesion and structural preservation rather than as an absolute improvement in bonding efficiency. The predominance of mixed failure modes in L group specimens supports the notion that enhanced interfacial bonding may partially offset the reduction in mechanical strength, thereby contributing to the long-term reliability of zirconia restorations.

Clinically, establishing a durable bond between zirconia and resin cement remains essential for the longevity of implant-supported and adhesive restorations. A minimum shear bond strength of 10–20 MPa has been suggested as sufficient for reliable restorations [32,33]. Although the conventional treatments in this study exceeded this threshold, clinical reports still describe debonding failures [34]. This observation suggests that achieving the minimum bond strength alone does not guarantee long-term reliability; stronger and more durable adhesion is necessary. Conventional surface treatments such as 10-MDP and APA are effective but remain susceptible to hydrolytic degradation and surface contamination. LST represents a promising alternative that can generate reproducible microtextures without leaving chemical residues. When appropriately optimized, LST may enhance micromechanical retention and interfacial energy while maintaining the intrinsic stability of zirconia.

This study had certain limitations. It was performed under in vitro conditions that do not fully replicate the oral environment, and only a single commercially defined laser surface texturing condition and zirconia compositions were evaluated. Further studies are required to refine LST parameters and to validate long-term bonding reliability under clinically simulated conditions. Within these limitations, LST improved interfacial bonding and surface integrity but resulted in a moderate reduction in flexural strength, particularly for 5Y-PSZ. Optimizing the laser parameters to achieve a balance between adhesion enhancement and mechanical stability may expand the clinical applicability of this technique.

## 5. Conclusions

Within the limitations of this in vitro study, both zirconia composition and surface treatment significantly affected bonding performance and mechanical behavior. Laser surface texturing (LST) yielded higher shear bond strength and greater surface roughness than conventional methods, demonstrating distinct micromechanical advantages. However, this improvement was accompanied by a decrease in flexural strength, particularly in 5Y-PSZ, underscoring the importance of maintaining an appropriate balance between adhesion and mechanical resilience. Optimization of laser parameters is therefore essential to enhance interfacial bonding while minimizing the adverse effects on structural integrity.

## Figures and Tables

**Table 1:** Two-way ANOVA results for surface roughness (Ra), shear bond strength (SBS), and biaxial flexural strength (BFS).

Two-Way ANOVA	Source	df	Sum of Squares	Mean Square	F	*p* Value
Surface roughness (Ra)	Material (3Y vs. 5Y)	1	0.0107	0.0541	0.0395	0.05
	Treatment (A/P/L)	3	350.7187	117.62	430.9404	<0.05
	Material × Treatment	3	0.0172	0.037	0.0211	0.84
	Residual	56	15.1918	0.136	—	
Shear bond strength (SBS)	Material (3Y vs. 5Y)	1	0.146	0.146	0.007	0.93
	Treatment (A/M/P/L)	3	8605.116	2868.372	138.091	<0.05
	Material × Treatment	3	425.895	141.965	6.835	<0.05
	Residual	72	1495.555	20.772		
Biaxial flexural strength (BFS)	Material (3Y vs. 5Y)	1	2,028,929	2,028,929	87.93	<0.05
	Treatment (A/P/L)	2	1,176,088	588,044	25.49	<0.05
	Material × Treatment	2	117,262	58,631	2.54	0.09
	Residual	54	1,245,958	23,074		

**Table 2:** Elemental composition (wt%) of zirconia surfaces according to surface treatment.

Group	O (wt%)	Y (wt%)	Zr (wt%)
3Y-A	29.65	6.52	63.83
5Y-A	32.49	6.33	61.18
3Y-M	36.41	5.66	57.93
5Y-M	29.53	6.17	64.30
3Y-P	32.23	6.52	61.25
5Y-P	34.89	5.97	59.14
3Y-L	35.75	3.51	60.70
5Y-L	37.18	5.67	57.15

A: As-milled, M: 10-methacryloyloxydecyl dihydrogen phosphate, P: Airborne particle abrasion, L: laser surface texturing.

## Data Availability

The raw data supporting the conclusions of this article will be made available by the authors on request.
